# Punctuality

**Published:** 1881-04

**Authors:** 


### PUNCTUALITY.

It is astonishing how many people there are who neglect punctuality, and thousands have failed in life from this cause alone; it is not only a serious vice in itself, but it is the fruitful parent of many other vices, so that he who becomes the victim of it gets involved in toils from which it is almost impossible to escape. It makes the merchant wasteful of time; it saps the business reputation of lawyers, and it injures the prospect of the mechanic, who might otherwise rise to fortune; in a word, there is not a profession, not a station in life, which is not liable to the canker of the destructive habit. It is a fact not always remembered, that Napoleon's great victories were won by infusing into his subordinates the necessity of punctuality to the minute. It was his plan to manœuvre over large spaces of country, so as to render the enemy uncertain where he was about to strike a blow, and then suddenly to concentrate his forces and fall with irresistible power on some weak point of the extended lines of the foe. The execution of this system demanded that each division of the army should arrive at the specified time punctually; for, if any part failed to come up, the battle was lost. It was by imitating this plan that the allies finally succeeded in overthrowing the emperor. The whole Waterloo campaign turned on these tactics. At Mt. St. Jean Blucher was punctual, while Grouchy was not; and the result was that Napoleon fell and Wellington triumphed.

In mercantile affairs punctuality is as important as in military. Many are the instances in which the neglect to renew an insurance punctually has led to serious loss. With sound policy do the banks insist, under the penalty of a protest, on the punctual payment of notes, for were they to do otherwise, commercial transactions would fall into inextricable confusion. Many and many a time has the failure of one man to meet his obligations brought on the ruin of a score of others, just as the toppling down in a line of bricks of the master brick causes the fall of all the rest. Thousands remain poor all their lives, who, if they were more faithful in their word, would secure a large run of custom, and so make their fortunes. Be punctual if you would succeed.

---

Col. Ingersoll says: "To me the future is exceedingly bright" And yet it is said the Colonel doesn't believe in the Bible, where his future is graphically described. A lake of fire is always bright. It is also hot.—*Norristown Herald.*

